# An Integrated Chemiluminescence Microreactor for Ultrastrong and Long‐Lasting Light Emission

**DOI:** 10.1002/advs.202000065

**Published:** 2020-06-17

**Authors:** Zhenyu Xiao, Yutong Wang, Ben Xu, Shunfu Du, Weidong Fan, Dongwei Cao, Ying Deng, Liangliang Zhang, Lei Wang, Daofeng Sun

**Affiliations:** ^1^ College of Science School of Materials Science and Engineering China University of Petroleum (East China) Qingdao Shandong 266580 P. R. China; ^2^ Key Laboratory of Eco‐Chemical Engineering Taishan Scholar Advantage and Characteristic Discipline Team of Eco‐Chemical Process and Technology College of Chemistry and Molecular Engineering Qingdao University of Science and Technology Qingdao 266042 P. R. China

**Keywords:** chemiluminescence, controlled diffusion, metal–organic frameworks, microreactors

## Abstract

A porous metal–organic framework [Ba(H_2_ **L^LOMe 2−^**)·DMF·H_2_O]·2DMF (**UPC‐2**) (H_4_ **L^LOMe^** = 4ʹ,4ʹʹʹ‐(2,3,6,7‐tetramethoxyanthracene‐9,10‐diyl)bis([1,1ʹ‐biphenyl]‐3,5‐dicarbo‐xylic acid N,N‐Dimethylformamide [DMF]), which can act as an excellent chemiluminescence microreactor, is designed and constructed. In the framework of **UPC‐2**, the catalytic Ba cluster and electron‐rich anthracene fluorescent centers are fixed and interconnected in an orderly fashion, and this can shorten the energy transfer path and weaken the relaxation of the chemiluminescence process. Meanwhile, the rhombic channels of **UPC‐2** can provide a proper diffusion ratio of reactants to support a stable and continuous energy supply. The **UPC‐2** chemiluminescence microreactor exhibits an ultrastrong and long‐lasting light emission, which possesses potential application in emergency lights and biological mapping. The concept of the chemiluminescence microreactor and its construction using a metal–organic framework as a platform will promote further research in the design and fabrication of functional MOFs for chemiluminescence applications.

## Graphical abstract

To enhance the light‐emitting intensity and extend the lasting time of chemiluminescence (CL) reaction, a concept of “integrated chemiluminescence microreactor” is proposed and constructed using a metal–organic framework as a platform. The as‐obtained microreactor presents an ultrahigh light‐emitting intensity which could be observed by the naked eye even after 540 min.

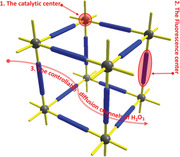

Chemiluminescence (CL), a kind of cold light generated from chemical reactions, has been widely investigated in recent decades.^[^ ^1^ ^]^ Due to its high light emission efficiency, ease of operation, and outstanding monochromaticity, CL has been widely applied in emergency lights,^[^ ^2^ ^]^ biosensors,^[^ ^3^ ^]^ gene reporters, and bioimaging.^[^ ^4^ ^]^ Especially for bioimaging field, the CL process is recognized as one of the most effective ways to track and monitor the biological reactions in real‐time.^[^ ^5^ ^]^ For example, the early detection of viscera disease can be achieved by Pu's group through real‐time CL imaging.^[^ ^6^ ^]^ The aforementioned applications of CL reaction commonly require a strong and constant light emission to offer a low detection limit and high sensor sensitivity.^[^ ^7^ ^]^ However, strong and stable CL emission is still uncommon and most CL systems present flash‐type light emission, which hinders the further applications of the CL system.^[^ ^8^ ^]^

CL can be viewed as a chemically initiated electron exchange luminescence (CIEEL), as has been proposed by Schuster.^[^ ^9^ ^]^ The CL process can be broken down into three steps: (i) a catalytic process in which OH^•^ and O_2_^•−^ radicals are formed by the catalytic decomposition of H_2_O_2_;^[^ ^10^ ^]^ (ii) an energy transfer process in which electron and energy transfer between a radical and an organic ligand occurs to produce an excited chromophore F*;^[^ ^11^ ^]^ and (iii) a light emission process in which the F* reverts to the ground state with the emission of light.^[^ ^12^ ^]^ In a commonly reported solvent CL system (**Figure** **1**a), the catalytic centers in the solutes catalyze the decomposition reaction of H_2_O_2_ to provide OH^•^ and O_2_^•−^ transfer path and the relaxation phenomenon of the free fluorescence center limit the light‐emitting intensity.^[^ ^13^ ^]^ Meanwhile, due to the fast diffusion ratio of H_2_O_2_ in solution and the high catalytic efficiency of the metal centers in the solutes, the H_2_O_2_ will be consumed rapidly and as a consequence, the solvent system normally presents weak and flash‐type light emission. Although it is reported that the electron‐rich and uncoordinated carboxylate group can stabilize radicals to provide higher light‐emitting intensity,^[^ ^14^ ^]^ this fails to solve the basic problems of the solvent system. Recently, a functional cluster system was developed, as shown in Figure 1b. Employing covalent, coordination, or other weak interactions, the free luminescence centers are fixed on the surface of the catalytic center to form a functional cluster, which dramatically shortens the energy transfer path, and weakens the relaxation of luminescence center.^[^ ^15^ ^]^ In this way, the light‐emitting intensity is significantly improved, but the lasting time is still limited as a result of the fast and uncontrollable diffusion ratio of the reaction medium. As an extension of the cluster strategy, the concept of a “functional microreactor” will be the next generation of CL systems due to it can not only take the advantage of the cluster system, but can also offer a proper H_2_O_2_ diffusion ratio to support a continuous energy supply, as shown in Figure 1c. Following this concept, Liu et al. and Li et al. report the macroscopical integration of catalytic center and fluorescence center in hydrogel (controlled‐diffusion strategy) and tablet (controlled‐release strategy) platform, and strong and long‐lasting light emission are achieved.^[^ ^7a,c^ ^]^ Therefore, the rational choice of a platform upon which to construct a CL microreactor, is a pathway to greatly improved CL.

**Figure 1:**
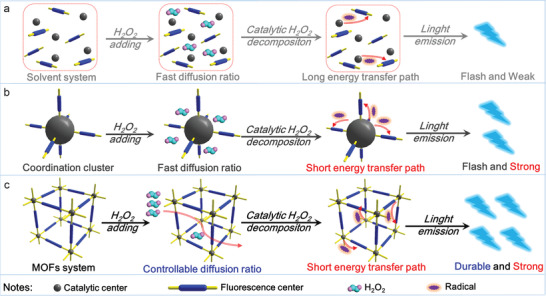
Three kinds of chemiluminescence strategy: a) solvent system; b) cluster system; c) microreactor system.

Metal–organic frameworks (MOFs), a new type of functional porous materials, have received much attention from chemists, due to their high surface area and tunable framework, as well as their potential applications in gas storage and separation, catalysis, luminescent probes, and drug delivery.^[^ ^16^ ^]^ Since MOFs are built from organic linkers and metal ions or clusters, the design and fabrication of MOFs as microunits with expected functions is plausible.^[^ ^17^ ^]^ Compared with the well‐reported semiconductor nanoparticles, metal nanoparticles (Ag, Au, and alloy), carbon dots, mesoporous silica, and porous materials (zeolite, organic polymer, and hierarchical composite), MOFs fit perfectly the requirements of a CL microreactor in that the catalytic and fluorescence centers are fixed in a precise framework with an accuracy of atom/molecule level.^[^ ^18^ ^]^ However, CL of MOFs is in its infancy. Xie et al. reported the first direct CL Cd‐MOF using an anthracene ligand as the fluorescence center, but the emission intensity is unsatisfactory due to its narrow channels.^[^ ^19^ ^]^

It is also reported that MOF can be used as a platform to loading or capping of chemiluminescence‐inducing species, but these systems present more uncertainty due to the random distribution of involved molecules.^[^ ^7^, ^12^, ^20^ ^]^ Therefore, the construction of MOFs based CL microreactor with precise atom/molecule location and controllable diffusion channels as well as the further optimization of each of the functional groups (the catalytic center, the fluorescence center, and the pore size) are still worthy for further exploration.

In this work, we synthesized a new electron‐rich carboxylate ligand H_4_ **L^LOMe^**(4ʹ,4ʹʹʹ‐ (2,3,6,7‐tetramethoxyanthracene‐9,10‐diyl)bis([1,1ʹ‐biphenyl]‐3,5‐dica‐rboxylic acid)) as the fluorescence center. The self‐assembly of H_4_ **L^LOMe^** with Ba^2+^ ions resulted in two new MOFs, **UPC‐2** ([Ba(H2**L^LOMe 2−^**)·DMF·H_2_O]·2DMF [N,N‐Dimethylformamide]) with 1D rhombic channels, and **UPC‐3** ([Ba_2_(**L^LOMe^**)·1.5H_2_O]·H_2_O) with constricted pores. Due to the integration of the Ba^2+^ catalytic center with an electron‐rich and uncoordinated carboxylate functionalized fluorescence center (H_2_ **L^LOMe 2−^**) and proper 1D rhombic channels, **UPC‐2** demonstrates ultra‐ strong CL intensity and long‐lasting direct CL. The light‐emitting intensity is about 20 times higher than that of **UPC‐3**, and 200 times higher than that of the reported **Cd‐MOF**.^[^ ^20^ ^]^ The success of this work may promote further research in the design and fabrication of MOF‐ based microreactors for enhanced chemiluminescence performances.

Single crystal X‐ray studies reveal that **UPC‐2** crystallizes in a cubic space group *Pnma*, and is a 3D porous framework based on 1D barium rod‐shaped SBUs. The asymmetric unit consists of a half Ba(II) ion, a half H_4_ **L^LOMe^** ligand, a half coordinated water molecule, a half coordinated DMF molecule, and one uncoordinated DMF molecule. The organic ligand of H_4_ **L^LOMe^** is partly deprotonated during the solvothermal reaction (**Figure** **2**a). The center Ba^2+^ ion is coordinated by nine oxygen atoms from H_2_ **L^LOMe 2−^** ligands, water and DMF molecules. Adjacent Ba^2+^ ions are connected by three oxygen atoms from carboxylate groups and DMF molecules to generate infinite Ba—O—Ba rods along the [1 0 0] direction with the closest Ba···Ba distance of 4.486 Å, as shown in Figure 2c. The Ba–O–Ba rod‐shaped SBUs are stacked in parallel and linked by the backbone of H_2_L^LOMe 2−^ to give rise to 1D rhombic channels along the *a*‐axis with the dimensions of 13.01 × 50.16 Å (Figure 2e). PLATON program analysis of **UPC‐2** demonstrates that there is ≈33.8% solvent‐accessible volume (total: 5577.9 Å^3^). **UPC‐3** crystallizes in a monoclinic space group *I2/c*, and the asymmetric unit consists of two Ba(II) ions (Ba1 and Ba2), one H_4_L^LOMe^ ligand, one and a half coordinated water molecules, and one free water molecule. There are two kinds of connection modes between Ba1 and Ba2: one is connected by three oxygen atoms from carboxylate groups and water molecules; the other is bridged by one carboxylate group with μ_2_‐η1:η1 mode and one oxygen atom from carboxylate group. The adjacent Ba^2+^ ions are connected by the Ba1–Ba2–Ba1–Ba1–Ba2–Ba1–Ba2–Ba1–Ba1–Ba2 mode to form a ten‐membered ring, as shown in Figure 2d. The ten‐membered rings are connected to each other by sharing vertices or edges to form a 2D layer SBU in **UPC‐3**. The adjacent 2D layers are further bridged by **L^LOMe^** ^4−^, giving rise to the final 3D framework with constricted pores (Figure 2f). To explore the pore structure and BET surface area, the N_2_ adsorption/desorption curves of **UPC‐2** and **UPC‐3** were measured and showed that the desolvated **UPC‐2** exhibits the reversible Type‐I adsorption isotherms (Figure S1b, Supporting Information), suggesting the retention of the microporous structure after the removal of solvents from the crystalline sample. The uptake of N_2_ is 128.5 cm^3^ g^−1^ at 77 K and the BET surface area is 458.3 m^2^ g^−1^. In comparison, **UPC‐3** demonstrates a structure with constricted pores and a BET surface area of 0.99 m^2^ g^−1^ (Figure S2, Supporting Information).

**Figure 2:**
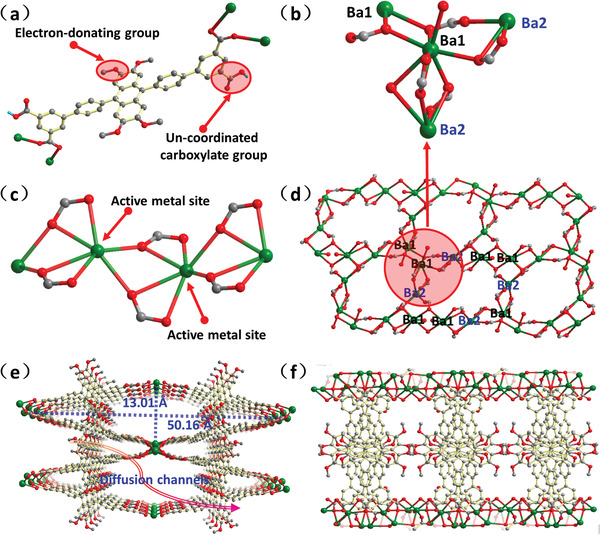
a) The coordination mode of the H_4_ **L^LOMe^** ligand in **UPC‐2**. b) The connection mode of Ba1 and Ba2 in **UPC‐3**. c) The 1D rod‐shaped SBU in **UPC‐2**. d) The 2D layer SBU in **UPC‐3**. e) Projection view of the framework **UPC‐2** along the *a*‐axis, showing a rhombic channels. f) Projection view of the framework **UPC‐3** along the *a*‐axis, showing constricted pores.

The structural character of **UPC‐2** fits well with the requirements of CL microreactor (accessible Ba center, electron‐rich, and carboxylate functionalized anthracene fluorescence center, and proper open channel), and direct CL of **UPC‐2** was determined by a traditional peroxyoxalate chemiluminescence (POCL) system.^[^ ^21^ ^]^ When crystals of **UPC‐2** (2 mg) are added into the POCL system, a brilliant blue light (*E*_m_ = 485 nm) can be observed with the naked eye (**Figure 3**a,c). The brightness continuously increased during the first 50 min and then gradually weakened due to the consumption of the chemiluminescence‐inducing species (Figure 3b; Figure S3, Supporting Information).^[^ ^22^ ^]^ The light‐emitting CL process can be extended for a long time. Even after 540 min, the light emission could still be observed with the naked eye (Figure 3c). As a contrast, when Ba(NO_3_)_2_ was added, no obvious light emission could be detected due to the lack of a fluorescence center (Figure 3b; Figure S4, Supporting Information). When H_4_ **L^LOMe^** ligand was added to the system, producing a common solvent system, the intensity reaches a maximum in the first 20 min and then rapidly declines, as shown in Figure S5 in the Supporting Information. After 120 min, the CL emission is almost invisible. The CL process of **UPC‐2** was also compared with that of **UPC‐3**, which was constructed by Ba^2+^ and H_4_ **L^LOMe^** ligand but possesses constricted pores (Figure S6, Supporting Information) and with that of the reported chemiluminescent Cd‐MOF [Cd_2_L_2_(DMF)_2_]•3H_2_O (Figure 3a; Figure S7, Supporting Information). It was observed that the light‐emitting intensity of **UPC‐2** at 50 min is about 20 times higher than that of **UPC‐3** and 200 times higher than that of the Cd‐MOF. For **UPC‐3**, the light‐emitting intensity reaches a maximum in the initial 120 min and then declines slowly. This may be attributed to the constricted pores which reduce the rate of the catalytic decomposition of H_2_O_2_. Furthermore, a series of experimental parameters, including the concentrations of tert‐BuOH and bis[3,4,6‐trichloro‐2‐(pentylo‐ xycarbonyl)‐phenyl] oxalate (CPPO), have been optimized (Table S2 and Figures S8–S11, Supporting Information). As shown in Figure S12a in the Supporting Information, the maximum light intensity of entry 3 (180 mg CPPO) is stronger than that of entry 1 (the optimized reaction condition), but the average CL intensity is weaker. It was also observed that in entry 5, there is an emission intensity platform between 40 and 100 min (Figure S12b, Supporting Information), which suggests potential applications in bioassays and biosensors. The emission quantum yield of **UPC‐2** system was measured by a luminol standard,^[^ ^23^ ^]^ and a quantum yield of 4.1 × 10^–4^ E mol^−1^ was calculated which is competitive that compared with the reported system.^[^ ^24^ ^]^ Furthermore, powder X‐ray diffraction (PXRD) examination demonstrates that **UPC‐2** has excellent stability in the POCL system (Figure S1a, Supporting Information). These results demonstrate that the effective integration and functionalization of the MOF‐based microreactor are beneficial to the catalytic and energy transfer processes in the CL reaction for enhanced CL intensity.

**Figure 3:**
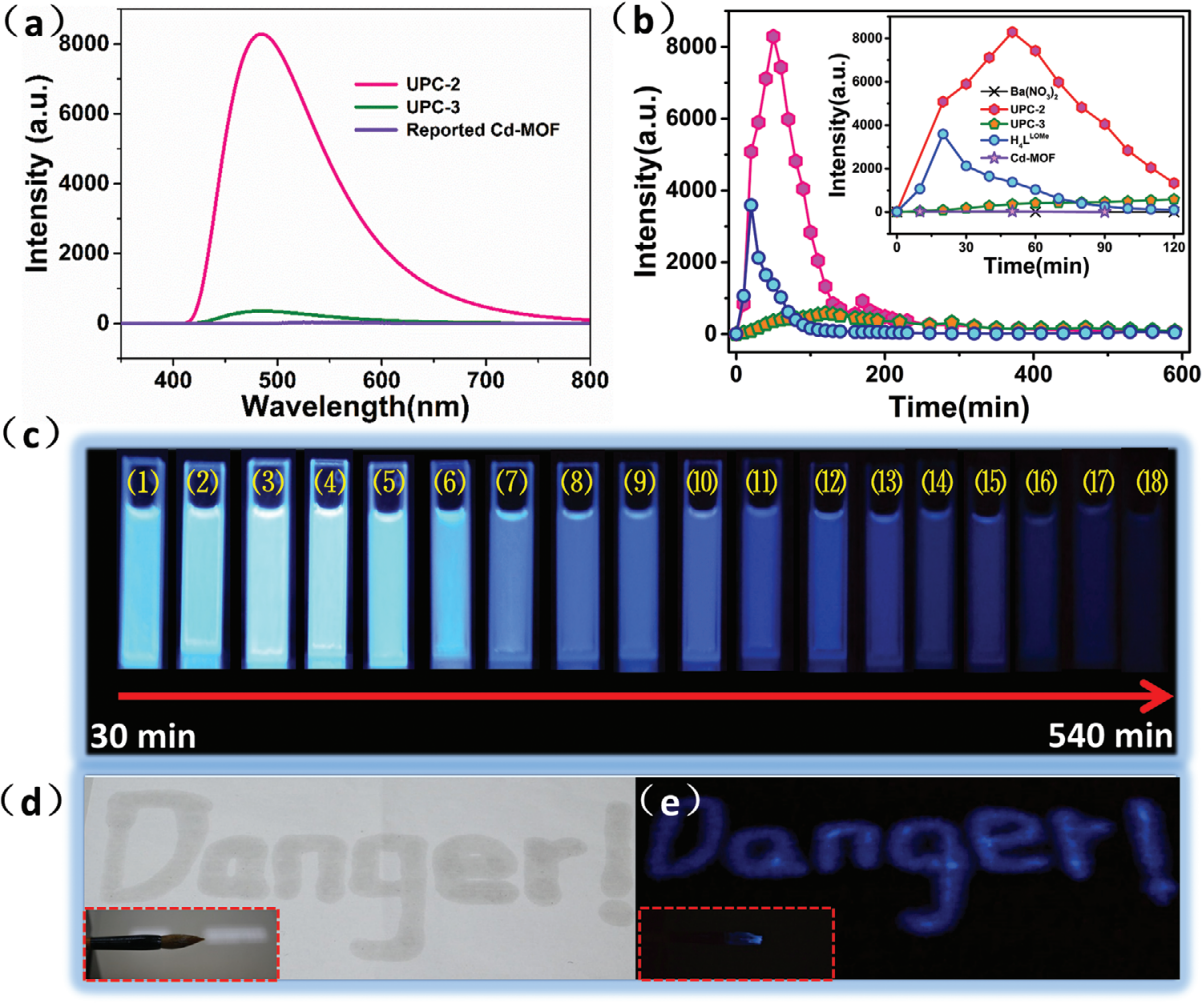
a) Chemiluminescence emission spectrum of **UPC‐2**, **UPC‐3**, and reported Cd‐ MOF. b) The CL intensity of **UPC‐2**, **UPC‐3**, **Cd‐MOF**, H_4_ **L^LOMe^** and Ba(NO_3_)_2_ as a function of reaction times. c) The changes in brightness of CL with the increasing time (numbers corresponding to the exact time of each inset figures, as 1:30, 2:60, 3:90, 4:120, 5:150, 6:180, 7:210, 8:240, 9:270, 10:300, 11:330, 12:360, 13:390, 14:420, 15:450, 16:480, 17:510, 18:540 min, respectively). The CL system of **UPC‐2** written mark and the insert Chinese brush under d) ambient light and e) in the dark.

The ultrastrong direct CL of **UPC‐2** prompted us to further study its potential as a light source. As is well known, CL materials can be used to fabricate cyalume, whose principal component is diphenyl oxalate. Generally, in cyalume, various dyes are used that can exhibit CL upon chemical energy stimuli. However, dye molecules are difficult to recover and degrade, and this leads to environmental pollution. **UPC‐2** is a crystalline powder and can be well dispersed in the liquid phase.^[^ ^25^ ^]^ Thus, it is easy to re‐collect **UPC‐2** by filtration, and this gives **UPC‐2** an advantage over dye molecules in cold light sources. After the addition of **UPC‐2** into a POCL system, paper can be written on using a Chinese brush (Figure 3d,e). The resulting mark emits blue light in the dark and thus has potential applications in warning signs.

To further explore the role of **UPC‐2** in the CL process, the measurements of electrochemical catalysis H_2_O_2_ were performed by cyclic voltammetry (CV) at a scan rate of 50 mV s^−1^. As shown in Figure S13 in the Supporting Information, a **UPC‐2@GC** electrode exhibited one obvious peak at about 0.815 V with 3 × 10^−3^ m H_2_O_2_ solution, while bare GC and **Cd‐MOF@GC** showed no electrochemical response. The electrocatalysis kinetics of a **UPC‐2@GC** electrode was studied by CV at different the scan rates (40–400 mV s^−1^, as shown in Figure S14a in the Supporting Information). A linear relationship between peak current intensity and the square root of scan rate was obtained (Figure S14b, Supporting Information), indicating a typical diffusion‐controlled reaction. These results show that H_2_O_2_ could diffuse in the channels of **UPC‐2** and be further decomposed via the catalysis of the Ba^2+^ center. Therefore, the CL mechanism of **UPC‐2** can be seen as a diffusion‐controlled CIEEL process (**Figure** **4**). H_2_O_2_ diffuses into the channel, then the Ba element and Ba–O group promote the decomposition of H_2_O_2_ molecule to form an OH^•^ radical.^[^ ^26^ ^]^ The radical makes contact with the H_2_ **L^LOMe^** ^2−^ ligand, and electron transfer occurs between H_2_ **L^LOMe 2−^** and the OH^•^ radical to form H**L^LOMe^** ^• 2−^ and H_2_O. The further reaction of OH^•^ radical with HO^–^ promotes the formation of O ^•−^, which further reacts with H**L^LOMe^** ^• 2‐^, resulting in the formation of H**L^LOMe^** ^3‐^. Then another CL emission reaction may occur between the explored –COO^−^ group of H**L^LOMe^** ^3−^ and O_2_^•−^ for the formation of –CO_4_^• 2−^, which can react with CPPO for further enhancement of the CL process.^[^ ^27^ ^]^

**Figure 4:**
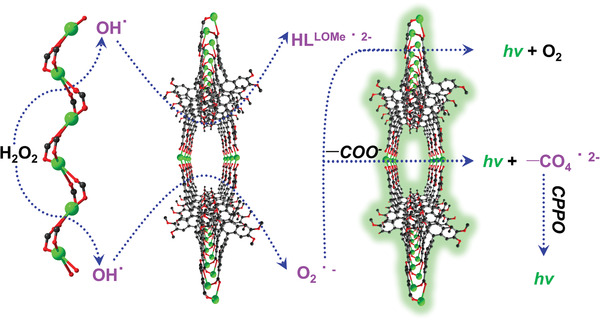
A possible mechanism for the POCL system.

In summary, a highly effective CL microreactor has been successfully constructed by integrating a catalytic center, a fluorescence center and the appropriate channels in a MOF (**UPC‐2**) platform. The coordination bonding of the catalytic group and a strong fluorescence center can shorten the energy transfer path for enhanced CL intensity, and the 1D open channels can control the diffusion ratio of reaction agents which enhance CL duration. Therefore, a light blue CL emission can be observed with the naked eye, and the lifetime can be longer than 540 min. In view of the merits of the functional CL microreactor, we believe that this work will facilitate the future development of the design and construction of MOFs for CL applications.

[CCDC 1875295 (**UPC‐2**) and 1946585 (**UPC‐3**) contains the supplementary crystallographic data for this paper. These data can be obtained free of charge from The Cambridge Crystallographic Data Centre via www.ccdc.cam.ac.uk/data_request/cif.]

## Conflict of Interest

The authors declare no conflict of interest.

## Supporting information

Supporting Information
