# Supplementary material for: An Integrated Chemiluminescence Microreactor for Ultrastrong and Long‐Lasting Light Emission
Source: Adv Sci (Weinh). 2020 Jun 17;7(15):2000065. doi: 10.1002/advs.202000065 (PMC7403964; doi:10.1002/advs.202000065)
Supplement: Supplementary file 1 — Supporting Information [file ADVS-7-2000065-s001.pdf]

© 2020 Wiley-VCH GmbH

ADVANCED SCIENCE
Open Access

Supporting Information

for *Adv. Sci.*, DOI: 10.1002/advs.202000065

An Integrated Chemiluminescence Microreactor for Ultra-Strong and Long-Lasting Light Emission

*Zhenyu Xiao#, Yutong Wang#, Ben Xu, Shunfu Du, Weidong Fan, Dongwei Cao, Ying Deng, Liangliang Zhang, Lei Wang and Daofeng Sun**

((Supporting Information can be included here using this template))

Copyright WILEY-VCH Verlag GmbH & Co. KGaA, 69469 Weinheim, Germany, 2020.

Supporting Information

An Integrated Chemiluminescence Microreactor for Ultra-Strong and Long-Lasting Light Emission

*Zhenyu Xiao[#], Yutong Wang[#], Ben Xu, Shunfu Du, Weidong Fan, Dongwei Cao, Ying Deng, Liangliang Zhang, Lei Wang and Daofeng Sun**

1. Materials and Characterization.

1.1 Material characterizations: Commercially available reagents were used as received without further purification. Barium nitrate ($Ba(NO_3)_2$, 99.5%, Energy Chemical), 4-Bromobenzaldehyde (C_7H_5BrO, 98%, Energy Chemical), 1,2-Dimethoxybenzene ($C_8H_{10}O_2$, 99%, Energy Chemical), Dibutyl phthalate ($C_{16}H_{22}O_4$, 99.5%, Energy Chemical), Bis(2,4,5-trichloro-6-carbopentoxyphenyl) oxalate ($C_{26}H_{24}Cl_6O_8$, 98%, Energy Chemical), Tetrakis(triphenylphosphine)palladium ($C_{72}H_{60}P_4Pd$, 99%, Pd 9% Energy Chemical) and tert-Butanol ($C_4H_{10}O$, ≥98.0%, Sinopharm Chemical Reagent Co.,Ltd). Dimethylformamide (DMF) and 1,4-Dioxane was freshly distilled from dry $MgSO_4$ and CaH_2 under nitrogen. X-ray powder diffractions patterns of the as-prepared samples were collected on a Bruker AXS D8 Advance instrument Cu-Kα radiation (λ = 1.5418 Å). The Brunauer-Emmett-Teller (BET) method was used to calculate the specific surface area of samples by N_2 adsorption-desorption measurement on a surface area analyzer ASAP-2020. ^1H NMR spectra were measured on a Bruker AVANCE-300 NMR Spectrometer. CL spectra were measured on an F-280 fluorescence spectrophotometer.

1.2 The synthesis process

Synthesis of H_4L^{LOMe} ligand:

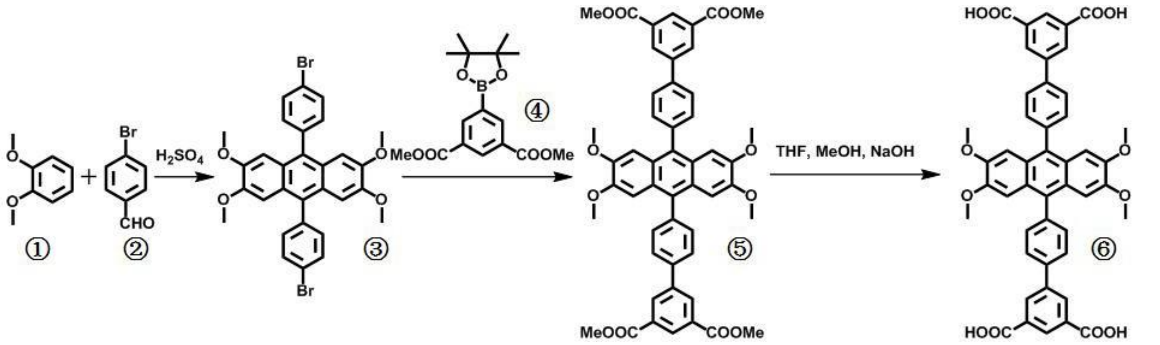

Synthesis of ③: ① (12.92 g) and ② (6.9 g) were dissolved in 80 mL CH_3COOH and 10 mL MeOH, and the mixture was cooled to 0 ℃. Then 80 mL H_2SO_4 was dropwise added in the mixture, and the mixture was kept at 0 ℃ for 5 days. After reaction, the mixture was poured in to 250 mL ice water, and a lots of solid was obtained. The resultant solid was dissolved in CH_2Cl_2, and ammonium hydroxide ($NH_3{\cdot}H_2O$) was added to adjust pH to neutral. A light yellow power was obtained after removed the organic solvent, which was further purified by column chromatography with CH_2Cl_2 as the eluent. ^{1}H NMR (400 MHz, $CDCl_3$): δ = 3.76(s, 12H), 6.75(s, 4H), 7.35(d, 4H), 7.74(d, 4H).

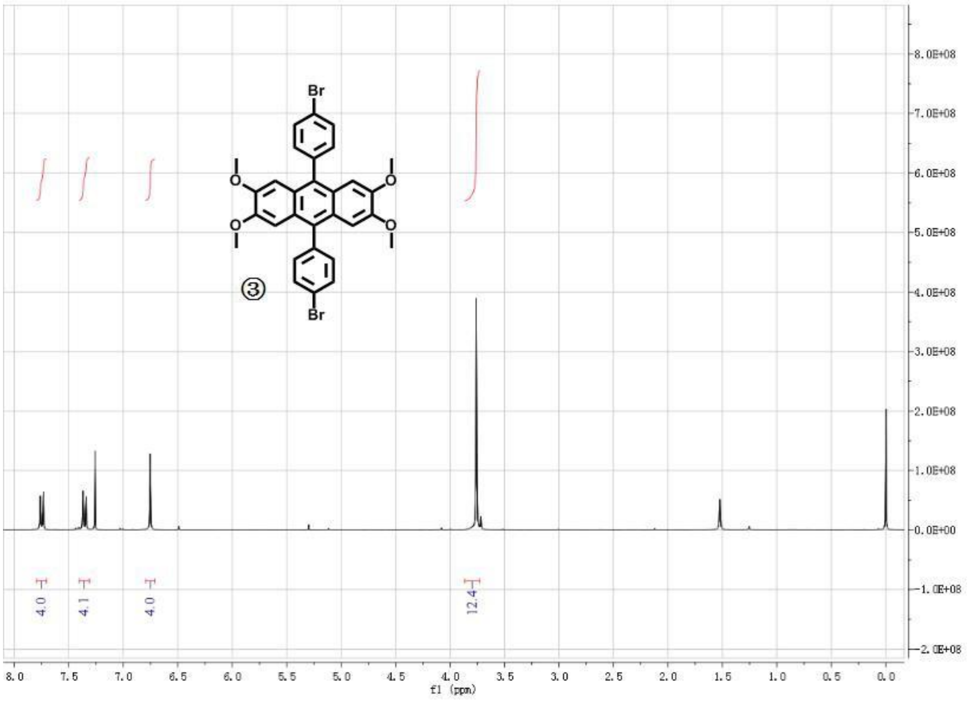

Synthesis of ⑤: ③ (3.2 g), ④ (4.64 g), and K_3PO_4 (8 g) were degassed and filled with nitrogen, which was repeated for three times. 1,4-dioxane (160 mL) was added with a syring under nitrogen. After this, $Pd(PPh_3)_4$ (0.2 g) was added to the stirred reaction mixture and the mixture was heated at 95 ℃ for 48 h under N_2 after which 1,4-dioxane was removed under a vacuum. The resultant solid was filtered and dried after refluxing in MeOH for 24 h. The resulting crude product was purified by column chromatography ($CHCl_3$:EtOAc = 60:1 as the eluent). ^{1}H NMR ($CDCl_3$): δ = 3.77 (s, 12H), 4.03 (s, 12 H), 6.88 (s, 4H), 7.64 (d, 4H), 7.96 (d, 4H), 8.67 (d, 4H), 8.73 (t, 2H) .

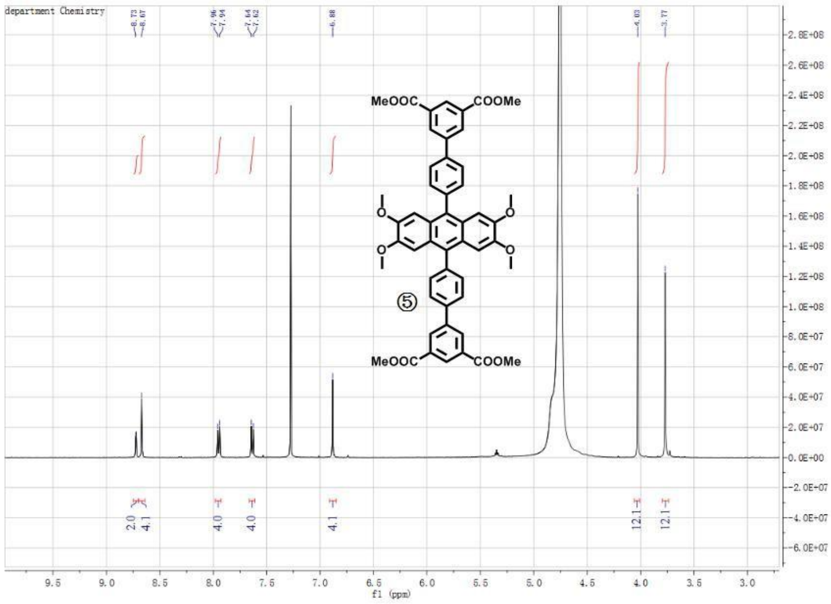

Synthesis of ⑥: ⑤ (1.5 g) was then suspended in a mixture of THF (50 mL) and MeOH (50 mL), to which 50 mL of 10 M KOH aqueous solution was added. The mixture was stirred overnight and the THF and MeOH were removed under a vacuum. Dilute HCl was added to the remaining aqueous solution until the solution was at pH = 2. The solid was collected by filtration, washed with water and MeOH, and dried to give the yellow solid ⑥ . ^{1}H-NMR (DMSO): δ = 3.63 (s, 12H), 6.96 (s, 4 H), 7.65 (d, 4H), 8.09 (d, 4H), , 8.59 (d, 4H), 13.4 (s, 3.8H).

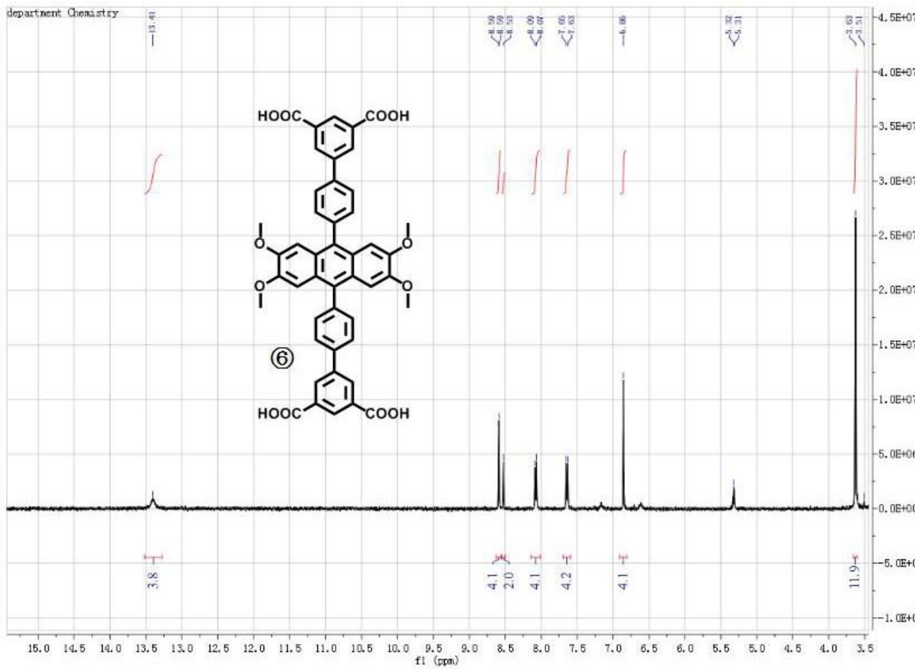

Synthesis of (2E,2'E)-3,3'-(anthracene-9,10-diyl)diacrylic acid H_2L ligand: The synthesis process of H_2L ligand was following our previous reported method.[2] After a *Heck* and hydrolysis reaction, the targe yellow power was obtained.

Synthesis of UPC-2: A mixture of $Ba(NO_3)_2$ (20 mg, 0.07 mmol) and $\mathbf{H_4L^{LOMe}}$ (5 mg, 0.006 mmol) was dissolved in DMF/H_2O mixed solvents (4 ml v/v 3:1). Then, the solution was poured into a 10 ml vial, kept at 90 ^{o}C for 24 hours. The light yellow crystals were collected and dried in air (yield: 70% , based on organic ligand).

Synthesis of UPC-3: The synthesis process of **UPC-3** is similar to the process of **UPC-2**, but the mixture solution was sealed in a Teflon-lined stainless container and reacted at 130°C for 24 hours.

Synthesis of Cd-MOF: The synthesis process is similar to the reported process,[1] $Cd(NO_3)_2$•4H_2O (920 mg, 3 mmol) and H_2L (100 mg, 0.3 mmol) were added in a mixture solution of DMF/H_2O (320 ml v/v 5:3), and the mixture was heated at 65°C for 7 days. After cooling to room temperature, the yellow $[Cd_2L_2(DMF)_2]$•3H_2O crystal was obtained and named as Cd-MOF.

Table S1. crystallographic data and refinement parameters of **UPC-2** and **UPC-3**

Compound	**UPC-2**	**UPC-3**
Empirical formula	$C_{56}H_{55}BaN_3O_{16}$	$C_{46}H_{32}Ba_2O_{15}$
Formula weight	1163.2	1099.4
Crystal system	Orthorhombic	Monoclinic
Space group	Pnma	I2/c
a/Å	8.52(3)	16.8785(6)
b/Å	50.09(14)	13.3406(3)
c/Å	12.96(8)	46.7943(17)
α/°	90.00	90.00
β/°	90.00	95.604(4)
γ/°	90.00	90.00
Volume/Å^3	5527.6(4)	10486.3(6)
Z	4	8
ρ_{calc}mg/mm^3	1.37	1.39
mμ/mm^{-1}	6.18	12.1
F(000)	2292.0	4320.0
Index ranges	$-10 \leq h \leq 10$, $-44 \leq k \leq 59$, $-13 \leq l \leq 15$	$-16 \leq h \leq 20$, $-16 \leq k \leq 7$, $-57 \leq l \leq 49$
Reflections collected	12208	19832
Independent reflections	4995 [Rint = 0.0361, Rsigma = 0.0422]	9787 [Rint = 0.0267, Rsigma = 0.0401]
Data/restraints/parameters	4995/25/351	9787/0/570
Goodness-of-fit on F^2	1.080	1.085
Final R indexes [I>=2σ (I)]	$R_1 = 0.0735$, $wR_2 = 0.1695$	$R_1 = 0.0734$, $wR_2 = 0.2288$
Final R indexes [all data]	R1 = 0.0902, wR2 = 0.1822	$R_1 = 0.0934$, $wR_2 = 0.2662$
Largest diff. peak/hole /e Å^{-3}	0.93/-3.32	1.57/-1.76

$^a R_1 = \Sigma| |F_o|-|F_c| |/ \Sigma|F_o|$, $^b wR_2 = [\Sigma w(F_o^2-F_c^2)^2]/ \Sigma w(F_o^2)^2]^{1/2}$

1.3 X-ray crystallographic characterization: The X-ray intensity data of **UPC-2** and **UPC-3** was collected on an Agilent Technologies SuperNova single crystal diffractometer equipped with graphite monochromatic Cu Kα radiation (λ = 1.54184 Å) at 295K. With the help of Olex2,[3] the structure was solved with the Superflip[4] structure solution program using charge flipping and refined with the ShelXL[5] refinement package using least squares minimization. All nonhydrogen atoms were refined with anisotropic displacement parameters. The hydrogen atoms on the ligands were placed at idealized positions and refined by a riding model. The detailed crystallographic data and structure refinement parameters for these compounds are summarized in *Table S1*, ESI† (CCDC 1875295 and 1946585).

1.4 Chemiluminescence process: A typical procedure of chemiluminescence study like that CPPO (Bis(2,4,5-trichloro-6-carbopentoxyphenyl) oxalate, 140 mg), **UPC-2** ({[Ba($H_2L^{LOMe\ 2-}$)·DMF·H_2O]·2DMF}$_n$ (activated by dynamic vacuum at 100 °C for 3 h), 2 mg) and Tert-BuOH (tert-butyl alcohol, 0.4 mL) were added in 5 mL dibutyl phthalate solution. After stirring for 3 min, H_2O_2 (30% aqueous solution, 20 μL) was added in the prepared mixture. Then the mixture solution was detected by F-4600 fluorescence spectrophotometer with different reaction times. For other comparison test conditions, the related chemicals were replaced as following the *Table S2*. The instrument parameters are listed in *Table S3*. For the contrast experiment, the dosage of MOFs are changed to $Ba(NO_3)_2$ (1.0 mg), and a series of substitutions with similar moles of anthracene group, such as a mixture of $Ba(NO_3)_2$ (1.0 mg) and H_4L^{LOMe} (1.0 mg), **UPC-3** (1.9 mg) or **Cd-MOF** (0.9 mg).

Table S2. Results of the chemiluminescence study under different conditions..

	**UPC-2 (mg)**	**CPPO (mg)**	**Tert-BuOH (mL)**	**Max Intensity at related time (min)**
**1**	2	140	0.4	8288 at 50
**2**	2	60	0.4	5884 at 50
**3**	2	180	0.4	8935 at 50
**4**	2	2	0.2	6425 at 40
**5**	2	2	0.8	5644 at 50

Table S3. The instrument parameters of chemiluminescence process.

**Type**	**selection**
Light source	off
Scan mode	Emission
Scan speed	30000 nm min^{-1}
Data mode	Luminescence
EM Slit	20 nm
EM range	350-800 nm

***Measurement of the chemiluminescence quantum yield*:** The chemiluminescence quantum yield of **UPC-2** is determined by the literature method with the hemin catalyzed luminol and H_2O_2 system as a standard.[6,7] The standard system is performed by $1x10^{-4}$ M luminol and $6x10^{-2}$ M H_2O_2 in a sodium phosphate buffer (0.1 mol L^{-1}, pH = 11.6), and the chemiluminescence spectra were collected at every 10 seconds from 10 seconds after H_2O_2 was added to the reaction system. For **UPC-2** system, the equal amount of H_2O_2 was added to the mixture 3 ml solution of CPPO ($1x10^{-4}$ M) and **UPC-2** (0.5 mg), and the light emission

process was similar to the luminol system. Then the chemiluminescence quantum yield of **UPC-2** was calculated by the following equations:

$$\phi_{CL} = \frac{Q \times f_{lum} \times f_{photo}}{n} (einsteins / mol) \qquad \text{Eq. S1}$$

$$f_{lum} = \frac{\phi_{lum} \times n_{lum}}{Q_{lum}} \qquad \text{Eq. S2}$$

$$f_{photo} = \frac{f(\lambda_s)}{f(\lambda_{lum})} \qquad \text{Eq. S3}$$

where ϕ_{CL} is the chemiluminescence quantum yield of **UPC-2**, Q is the total light emission obtained by integration of emission intensity under time curves. f_{lum} is obtained by measuring the emission kinetics of the luminol reaction performed in standard conditions. f_{photo} is obtained from the sensitivity at the emission wavelength of the luminol standard, $f(\lambda_{lum})$, and the emission maximum of the **UPC-2**, $f(\lambda_s)$. n is the number of moles of luminol (n_{lum}) or the number of moles of CPPO (n).

1.5 Electrochemical measurement: Electrochemical experiments were carried out on a CHI760E electrochemical workstation in 0.1 M KOH solution at room temperature with a three electrode system: a platinum wire as the counter-electrode, a standard Hg/Hg_2Cl_2 electrode as the reference electrode, and the MOFs modified GC electrode as the working electrode.

***Fabrication of the working electrode*:** Prior to modification, the GC was first polished with 0.05 µm alumina powders on a polishing cloth to obtain a mirror-like surface. Then it was washed successively with 1:1 nitric acid, ethyl alcohol, and doubly distilled water in an ultrasonic bath and dried under a stream of nitrogen. MOFs could be cast on the surface of GC by dropping with 5 µL 1 mg mL^{-1} solution. After the electrode dried in air, 3 µL Nafion solution (0.5 wt%) was cast onto the surface of the MOFs modified GC electrode and dried at room temperature.

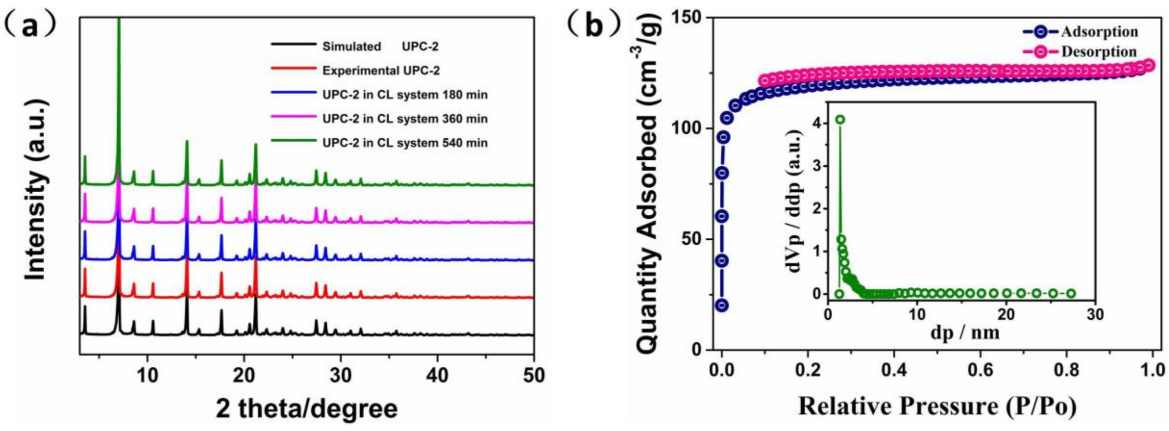

Figure S1. (a) The PXRD of simulated **UPC-2**, as-obtained **UPC-2** and re-collected **UPC-2** after CL with different times (180 min, 360 min and 540 min). (b) The N_2 adsorption isotherms at 77 K of **UPC-2** and the related pore size distribution (insert).

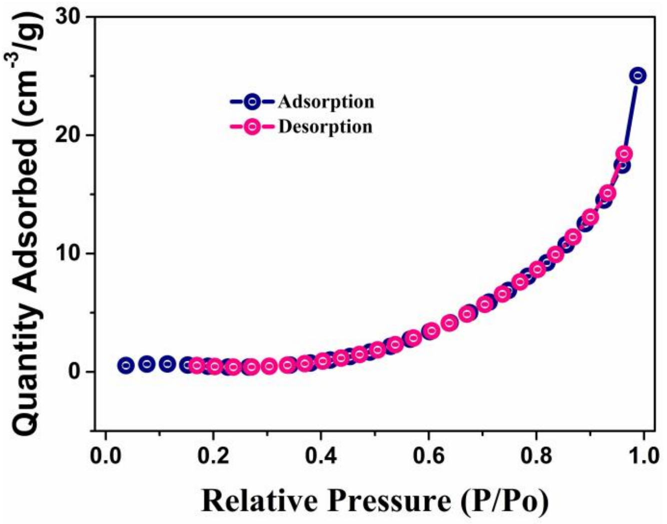

Figure S2. The N_2 adsorption isotherms at 77 K of **UPC-3**.

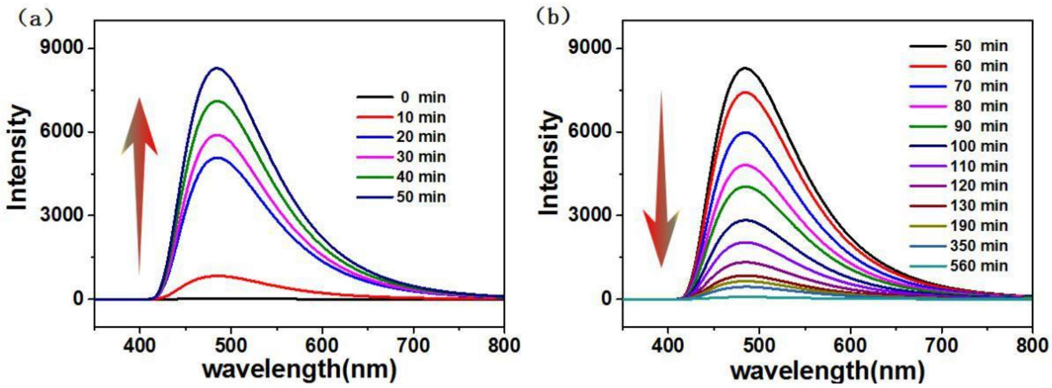

Figure S3. The CL emission spectra of **UPC-2**, (a) the increasing part of CL emission; (b) the decreasing part of CL emission.

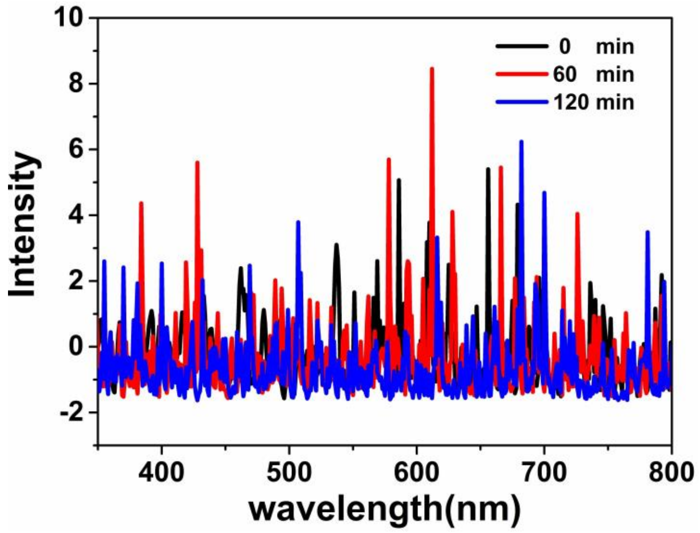

Figure S4. The CL emission spectra of $Ba(NO_3)_2$.

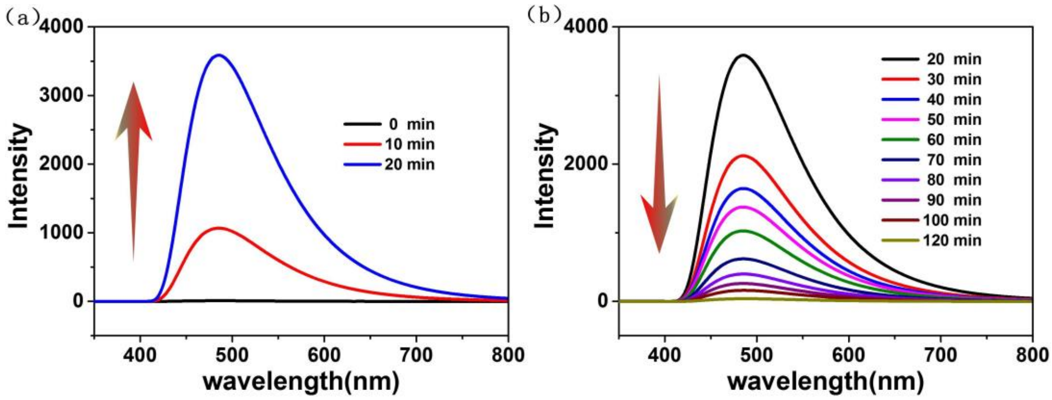

Figure S5. The CL emission spectrum of H_4L^{LOMe} under optimized condition, (a) the increasing part of CL emission; (b) the decreasing part of CL emission.

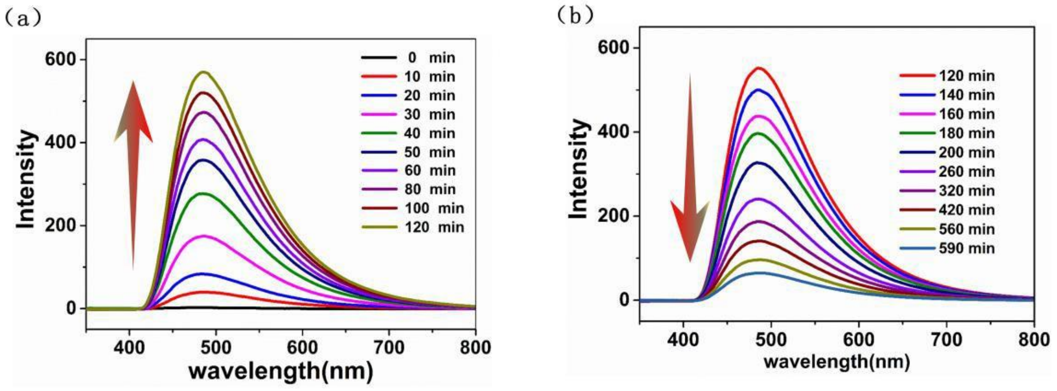

Figure S6. The CL emission spectrum of **UPC-3**, (a) the increasing part of CL emission; (b) the decreasing part of CL emission.

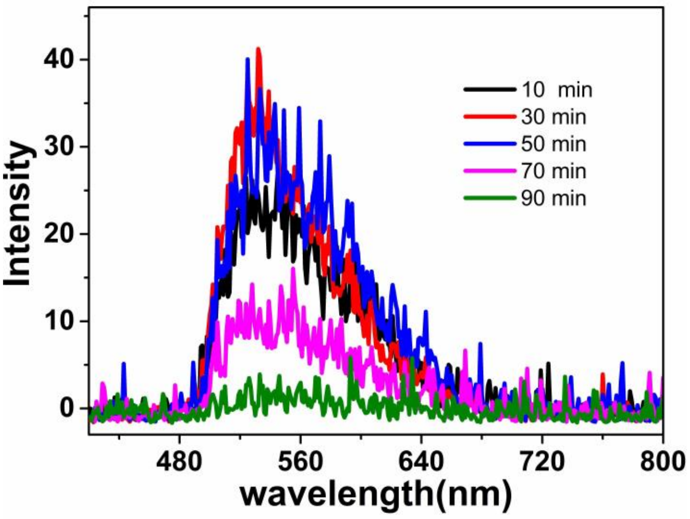

Figure S7. The CL emission spectrum of **Cd-MOF**.

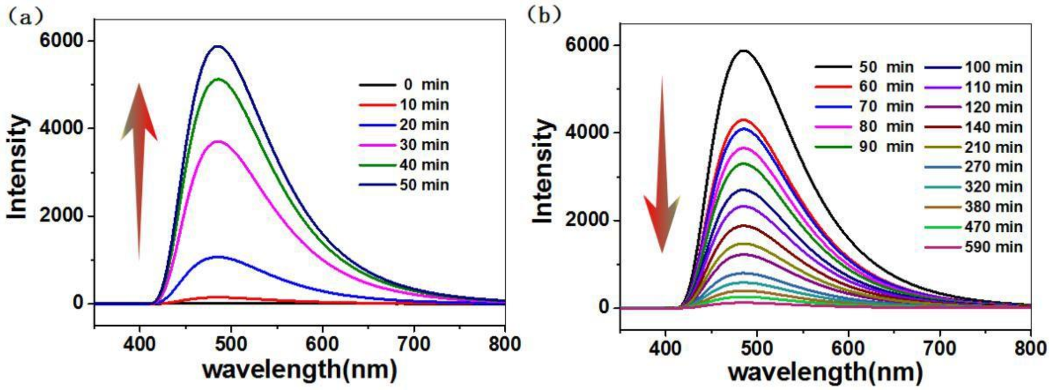

Figure S8. The CL emission spectrum of **UPC-2** under low CPPO condition (60 mg), (a) the increasing part of CL emission; (b) the decreasing part of CL emission.

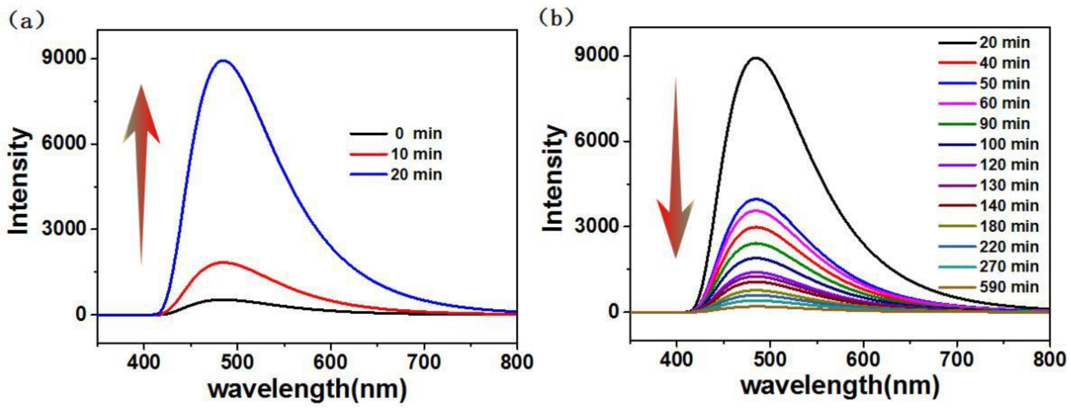

Figure S9. The CL emission spectrum of **UPC-2** under high Cppo condition (180 mg), (a) the increasing part of CL emission; (b) the decreasing part of CL emission.

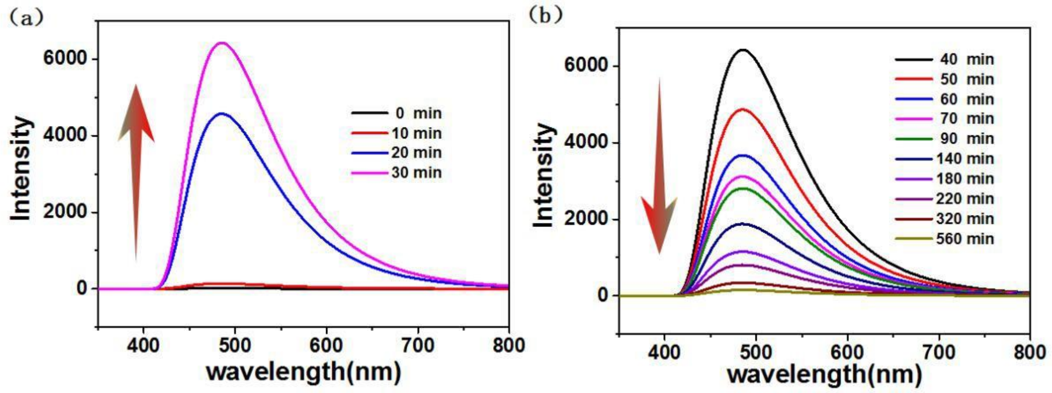

Figure S10. The CL emission spectrum of **UPC-2** under low Tert-BuOH condition (200 mL), (a) the increasing part of CL emission; (b) the decreasing part of CL emission.

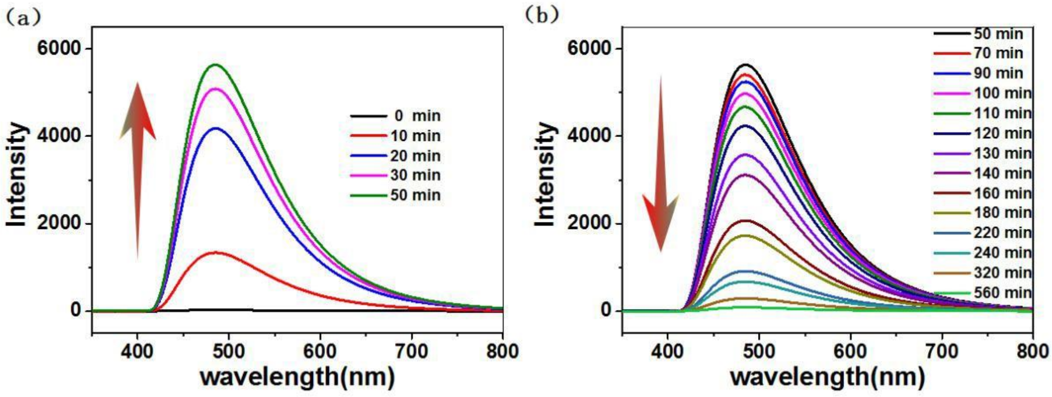

Figure S11. The CL emission spectrum of **UPC-2** under high Tert-BuOH condition (800 mL), (a) the increasing part of CL emission; (b) the decreasing part of CL emission.

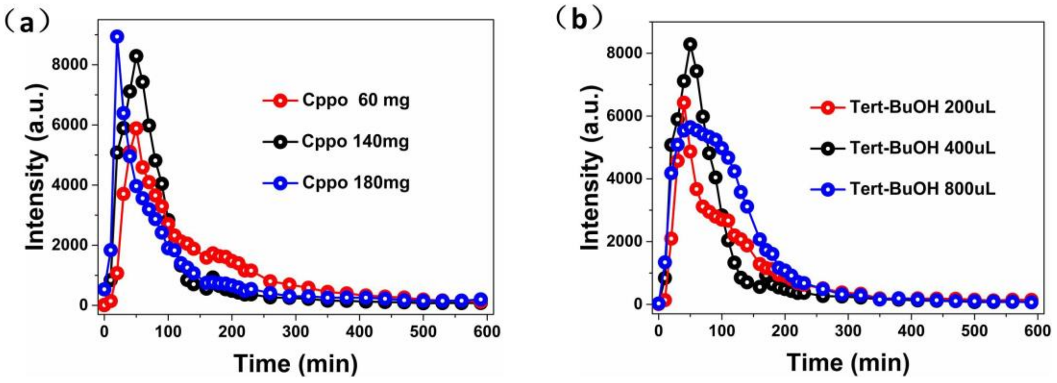

Figure S12. The CL intensity of **UPC-2** as a function of reaction times under different POCL conditions, (a) different contents of CPPO; (b) different contents of Tert-BuOH.

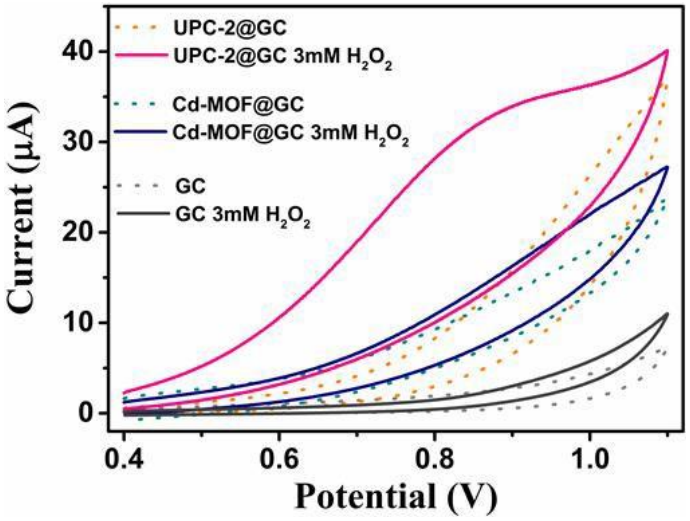

Figure S13. Cyclic voltammograms of GC, **Cd-MOF@GC**, **UPC-2@GC** electrodes in the absence (dotted line) and presence (solid line) of 3 mM H_2O_2 at scan rate of 50 mV s^{-1}.

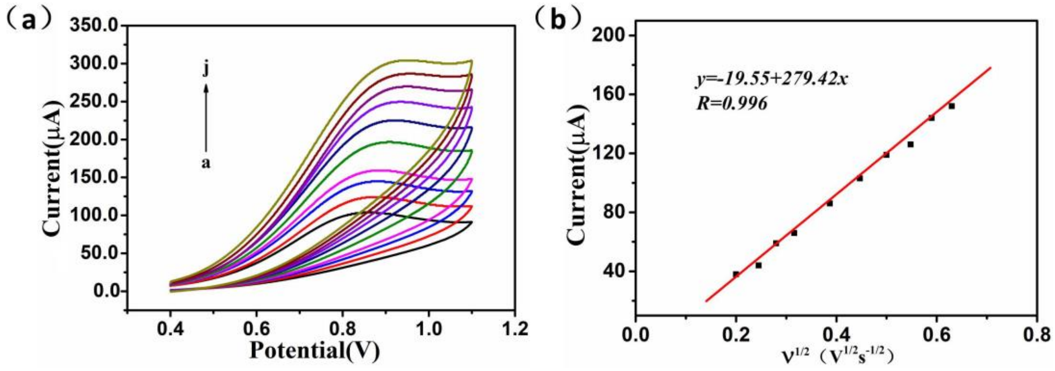

Figure S14. (a) CVs of the **UPC-2@GC** at different scan rates: 0.04, 0.06, 0.08, 0.10, 0.15, 0.20, 0.25, 0.30 0.35 0.4 V s^{-1}. (b) Plots of peak current vs. $v^{1/2}$ for the electrocatalysis of H_2O_2 at the **UPC-2@GC**.

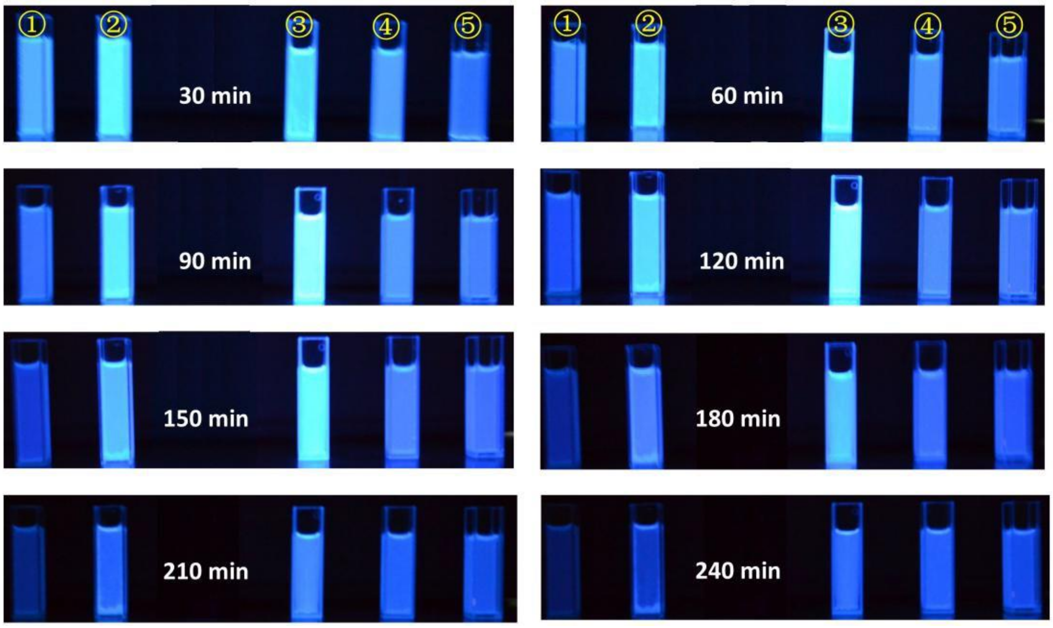

Figure S15. ① The brightness changes of CL of H_4L^{LOMe} with the increasing of times from 30-240 min under optimized condition. The brightness changes of chemiluminescence of H_4L^{LOMe} with the increasing of times from 30-240 min under different conditions, ② CPPO 180 mg; ③ optimized condition; ④ CPPO 60 mg; ⑤ Tert-BuOH 800 mL.

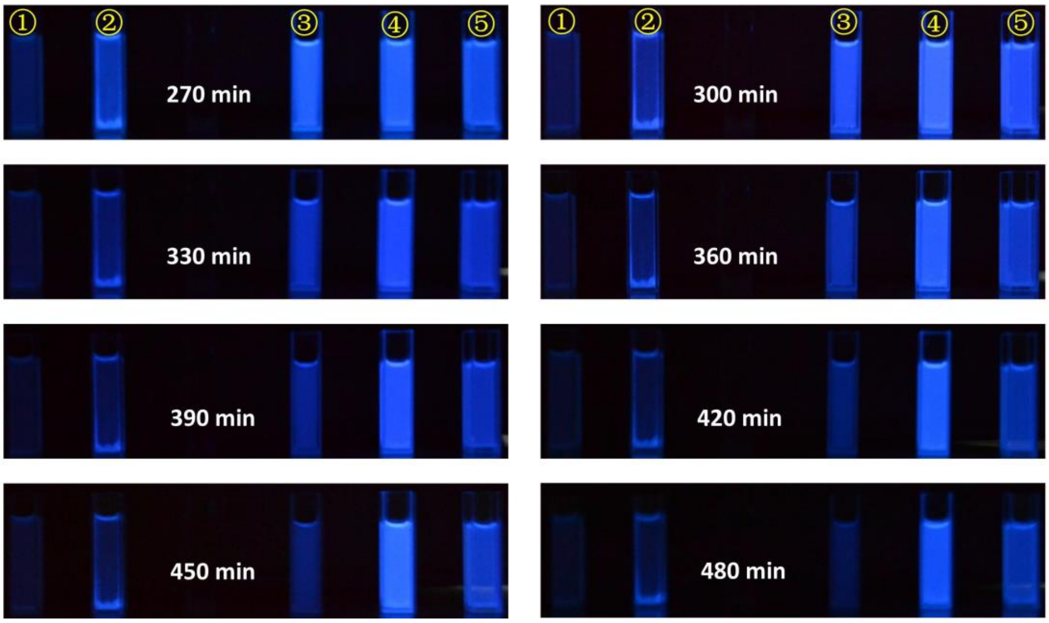

Figure S16. ① The brightness changes of chemiluminescence of H_4L^{LOMe} with the increasing of times from 270-480 min under optimized condition. The brightness changes of chemiluminescence of H_4L^{LOMe} with the increasing of times from 30-240 min under different conditions, ② Cppo 180 mg; ③ optimized condition; ④ Cppo 60 mg; ⑤ Tert-BuOH 800 mL.

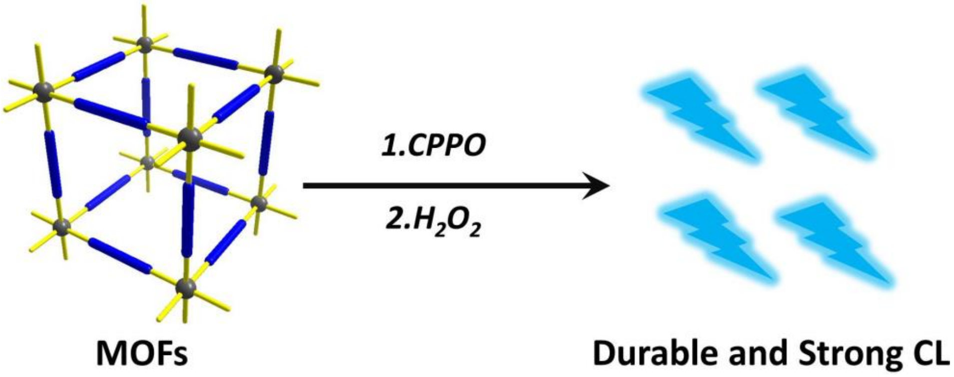

Figure S17. The brief diagram of the POCL system in this work.

References

[1] Yang, X. L. Chen, X. Hou, G. H. Guan, R. F. Shao, R. Xie, M. H. A Multiresponsive Metal-Organic Framework: Direct Chemiluminescence, Photoluminescence, and Dual Tunable Sensing Applications. *Adv. Funct. Mater.* **2016**, 26, 393-398.

[2] Wang, R. Liu, X. Huang, A. Wang, . Xiao, Z. Zhang, L. Dai, F. Sun, D. Unprecedented Solvent-Dependent Sensitivities in Highly Efficient Detection of Metal Ions and Nitroaromatic Compounds by a Fluorescent Barium Metal–Organic Framework. *Inorg. Chem.* **2016**, 55, 1782-1787.

[3] Dolomanov, O. V. Bourhis, L. J. Gildea, R. J. Howard, J. A. K. Puschmann, H. OLEX2: a Complete Structure Solution, Refinement and Analysis Program. *J. Appl. Cryst.* **2009**, 42, 339-341.

[4] Palatinus, L. Chapuis, G. Superflip- a Computer Program for the Solution of Crystal Structures by Charge Flipping in Arbitrary Dimensions. *J. Appl. Cryst*. **2007**, 40, 786-790.

[5] Sheldrick, G. M. A Short History of SHELX. *Acta Cryst. Sect. A: Found. Cryst.* **2008**, 64, 112-122.

[6] Zhen, X. Zhang, C. Xie, C. Miao, Q. Lim, K. L. Pu, K. Intraparticle Energy Level Alignment of Semiconducting Polymer Nanoparticles to Amplify Chemiluminescence for Ultrasensitive In Vivo Imaging of Reactive Oxygen Species *ACS nano*, **2016**, 10, 6400-6409.

[7] Felipe A. A. Glalci, A. S. Sergio, S. P. S. J. Muhammad, K. Wilhelm, J. B. Efficiency of Electron Transfer Initiated Chemiluminescence. *Photochem. Photobiol.*, **2013**, 89, 1299-1317.
